# Advancements in Nanocarrier Production Techniques and Methods for Enhanced Targeted Delivery of Drug: A Comprehensive Review

**DOI:** 10.2174/0115672018379860250722161458

**Published:** 2025-07-30

**Authors:** Atish Kumar S Mundada, Lokesh P Kothari, Kuldeep Vinchurkar, Saloni Yadav, Arprita Malhan, Mridul Guleria, Sudarshan Singh

**Affiliations:** 1 Department of Pharmaceutics, SNJBs SSDJ College of Pharmacy, Chandwad, Nashik, Maharashtra, 423101, India; £Present address: SVKM’s NMIMS School of Pharmacy & Technology Management, Indore, M.P., India;; 2 Department of Pharmaceutics and Pharmaceutical Technology, Krishna School of Pharmacy and Research (BITS Edu Campus), Dr. Kiran and Pallavi Patel Global University, Vadodara, Gujarat, 391240, India;; £ Present Address: Department of Pharmaceutics, Sandip Institute of Pharmaceutical Sciences (SIPS), Affiliated To Savitribai Phule Pune University (SPPU, Pune), Nashik, Maharashtra 422213, India;; 3 Acropolis Institute of Pharmaceutical Education and Research, Indore, Madhya Pradesh 453771, India;; 4 Department of Pharmaceutical Science, Guru Nanak Dev University, Amritsar, Punjab, 143005, India;; 5 Faculty of Pharmacy, Chiang Mai University, Chiang Mai, 50200, Thailand;; 6 Office of Research Administration, Chiang Mai University, Chiang Mai, 50200, Thailand

**Keywords:** Nanotechnology, nanocarriers, liposomes, niosomes, nano capsules, dendrimers

## Abstract

Nanotechnology has transformed drug delivery systems, leading to the creation of various nanocarriers that offer significant advantages over traditional methods. This review explores key techniques and methods for producing nanocarriers like liposomes, niosomes, dendrimers, nano-capsules, carbon nanotubes, polymeric micelles, and solid lipid nanoparticles. Operating within the nanoscale range (1-100 nm), these nanocarriers enhance drug efficacy, reduce side effects, and improve bioavailability. Liposomes are generated using methods, such as the Bangham procedure, solvent injection, and microfluidic channels. Nanocarriers have become fundamental to sophisticated drug delivery systems, providing improved precision, regulated release, and targeted therapeutic administration. Innovative methods, such as microfluidics and nanoprecipitation, have enhanced the scalability and consistency of nanocarriers, while progress in surface engineering, including ligand conjugation and stimuli-responsive coatings, facilitates improved targeting and controlled drug release. The advancement of biocompatible and biodegradable nanomaterials, including polymeric nanoparticles, liposomes, and dendrimers, has broadened the clinical utility of nanocarriers, especially in oncology, neurology, and gene therapy. This review underscores the versatility and potential of these nanocarriers in advancing drug delivery, emphasizing their capacity for targeted, efficient, and controlled therapeutic interventions.

## 1 INTRODUCTION

The prefix "nano" has become prevalent in numerous scientific disciplines and everyday life, to the extent that we are now living in a "nano" generation. This name for the chemical in consideration is as prevalent as the phrase "plastic." Numerous subjects within nanoscience and nanotechnology are perpetually acquiring new interpretations [1]. In addition to materials, items, or processes, firms such as Nanotero, NanoInk, and NanoSight utilize the "nano" prefix. Within the life sciences, gadgets, and imaging, "nano" particles and materials are revolutionizing different investigative and mechanical forms.

Previously, particles were identified and quantified in the micrometer range; however, the measuring focus has now transitioned to the nanoscale range, leading us to inhabit a "nano" world. Nanotechnology and nanoscience definitions have been hampered in several ways. According to the National Nanotechnology Initiative, nanotechnology is concerned with materials, their examination and manipulation in the size range of 1 to 100nm [2]. Due to various nanoscale size ranges, nanomaterials are distinct from their macro counterparts in terms of their altered properties and may unintentionally overcome typical biological barriers, sometimes posing threats to human health, like inhalation toxicity causes pulmonary edema, immunological adjuvant effects, systemic effects like blood coagulation, and cardiovascular side effects [3].

The progress made in the subject of nanotechnology in the last few years, particularly in the domain of drug delivery, is really notable. The applications of nanotechnology in the field of medicine, known as nanomedicine, have triggered the development of numerous types of drug-loaded nanocarriers like liposomes, niosomes, nanoparticles, dendrimers and micelles, among others. Fig. (**1**) illustrates the classification of the nanocarrier.

Currently, substantial efforts are focused on building nanoparticulate systems that can serve as useful diagnostic, therapeutic, or theranostics tools for serious diseases, such as cancer, infectious diseases, and neurological disorders [4]. In general, nanocarriers used for delivering the drug have an edge above traditional systems of drug delivery owing to the several potential benefits they offer. To start with, nanocarriers’ improved surface-to-volume ratio may permit for a greater medicate contact zone with the body at the same sedate concentration. The therapeutic results obtained was convincing with the same measurements at a lower dosage, and hence the drug's side effects and toxicity issues consequently reduced. Secondly, nanocarriers' surface chemistry can be tailored to specific medicines and targeted chemicals [5]. The ability to provide prolonged and sustained drug release is enabled by the tunable surface chemistry, ultimately improving the drug's bioavailability. Nanocarriers ensure availability of drug exactly where and when it’s required, with prolonged drug circulation than that of native forms of drugs in conventional delivering system. Moreover, these carriers protect the drugs from degradation by the surface chemical adjustment, permitting for a proficient route within the complex *in vivo* environment. The payload can also be directed not just to specific cell types for targeted delivery owing to the chemical change on the surface, but also to specific parts of the cell, allowing for improved intracellular drug trafficking. Moreover, nanocarriers can enable flexibility when it comes to drug formulation and medication administration routes [6].

The synergistic potential of combining nanotechnology with Traditional Chinese Medicine (TCM) to enhance the delivery and efficacy of natural therapeutic compounds. The authors identify several challenges inherent to TCM, such as poor water solubility, instability, and low bioavailability of active ingredients, which often hinder their therapeutic effectiveness. To address these issues, the integration of nanomedicine offers promising solutions.

1. Nanocarrier Systems: The study emphasizes the develop-ment of various nanocarriers—such as liposomes, dendri- mers, and polymeric nanoparticles—to encapsulate TCM-derived compounds. These nanocarriers can protect bioactive substances from degradation, enhance their solubility, and facilitate targeted delivery to specific tissues or cells, thereby improving therapeutic outcomes.
2. Enhanced Bioavailability: By employing nanotechno- logy, the bioavailability of natural products can be significantly increased. Nanocarriers enable controlled and sustained release of active compounds, ensuring a more consistent therapeutic effect and reducing the frequency of dosing.
3. Targeted Delivery: Functionalization of nanoparticles with sspecific ligands allows for precise targeting of diseased tissues, sminimizing off-target effects and enhancing the therapeutic index of sTCM compounds. This targeted approach is particularly beneficial in streating conditions where localized therapy is essential.
4. Challenges and Future Directions: The authors acknowledge several challenges in integrating nano- medicine with TCM, including regulatory hurdles, safety concerns, and the need for standardized protocols. They advocate for comprehensive research to establish the safety and efficacy of nano-TCM formulations and call for the development of regulatory frameworks to facilitate their clinical translation [7].

Therefore, this review underscores the versatility and potential of these nanocarriers in advancing drug delivery, emphasizing their capacity for targeted, efficient, and controlled therapeutic interventions. The literature was imported from various database, such as Scopus, Google Scholar, Web of Science, and ResearchGate, following exclusion of duplicate literature, and subsequently exported papers were further scrutinized for papers that are not linked with the theme of the paper specify considering the keywords. Furthermore, the exported papers were thoroughly checked for the information that were related to the objective of the review and required data was extracted for writing the review manuscript.

## 2 TARGETED DRUG DELIVERY

Nanocarriers are useful within the medicate conveyance preparation since they carry payload to the targets. Site-specificity has a significant therapeutic advantage as it avoids medications from being distributed to the incorrect locations. Chemotherapy medications can be exceedingly hazardous to normal and healthy human cells. Hence, it is expected that the therapeutic agent must get to the target tumor without spreading to other parts of the body. Nanocarriers provide the potential to enhance chemotherapy by mitigating its harmful, widespread effects on healthy, rapidly proliferating normal cells in the body. Nanocarriers can deliver medications to target site in four ways, and approaches used to achieve targeting can be deciphered from the terms used: Passive targeting, active targeting, pH dependent targeting, and temperature triggered targeting [7]. Fig. (**2**) depicts a schematic of drug-carrying nanoparticles being delivered to tumors either actively or passively to the targets [8].

## 3 PASSIVE TARGETING

The blood vessels that develop in a tumor cell contain numerous minuscule pores and thus a tumor is said to exhibit leaky vasculature. These pores not only allow the nanocarriers to enter, but also being trapped within the tumor due to poor lymphatic drainage [9]. This effect is termed as Enhanced Permeability and Retention (EPR) and was reported in 1986 by Matsumura and Maeda [10, 11]. Nanocarriers can thus extravasate into tumors through holes between endothelial cells and accumulate there. The ability of nanocarriers to reach the tumor's circulatory system, stuck and aggregate there, is referred to as passive targeting.

## 4 ACTIVE TARGETING

Active targeting entails decorating the surface of nanocarriers with targeting modules, like ligands or antibodies that exhibit specific affinity for the receptors and are over-expressed on malignant cells. Nanocarriers possess a significant surface-area-to-volume ratio that facilitates the loading of many ligands onto their surfaces. These targeting modules allow nanocarriers to be integrated directly into cells, although they come with significant disadvantages. However, non-specific binding to ligands configures nanocarriers slightly more hazardous, while positive charges on ligands may hamper the drug’s efficacy. Dynamic focusing can help to overcome the multi-drug resistance in tumor [12].

## 5 PH DEPENDENT TARGETING

pH dependent nanocarriers are designed to deliver the drugs in a certain range of pH through a trigger system. The basic mechanism involved in pH dependent targeting of nanocarriers involves the addition or removal of a proton from the nanocarriers, prepared using pH dependent macromolecule, results in significant adaptation change with the slightest or marginal change in the pH conditions in and around the delivering environment. The payload gets released in highly acidic conditions owing to the degradation of nanocarrier’s structure in that environment. However, these nanocarriers won't release payload in neutral or basic conditions, efficiently targeting malignant cells acidic surroundings while sparing normal body cells. This pH sensitivity could also be achieved by introducing copolymer chains, which have previously been shown to act in a pH-independent manner, in micelle systems [8]. These micelle-polymer complexes can avert the resistance to multiple drugs in tumor cells too. The micelle polymer releases the payload all at once rather than releasing it gradually under low pH. This rapid release mechanism helps in preventing the tumor from undergoing changes and become resistant to the treatment by cutting the time anticancer payload takes to destroy a tumor in half [13].

## 6 TEMPERATURE TRIGGERED TARGETING

At certain temperatures, some nanocarriers demonstrated to transport payloads more effectively [14]. The difference between tumor temperatures and the rest of the body’s temperatures acts as a security for tumor-specific location conveyance of these kinds of nanocarriers [15].

## 7 NANOCARRIERS

To improve the bioavailability of the drug, enhance drugs stability and achieve the targeting has been the focus of pharmaceutical research in recent years. Nanoparticulate drug delivery systems are in increasing demand as drug carriers owing to their minuscule size, enormous surface area and viable targetability. Nanocarriers could achieve the desired objectives like enhancing the efficacy of the drug along with decreasing its side effects and improving its physicochemical stability. Nanocarriers like liposomes, niosomes dendrimers, nano-capsules, carbon nanotubes, polymeric micelles, solid lipid nanoparticles, *etc*., have been prepared and assessed as carriers for a variety of drugs and for various routes. Fig. (**3**) depicts the various types of nanocarriers used in pharmaceuticals. Moreover, Table **1** illustrates various production techniques of various nanocarriers.

Nanocarriers are customizable in terms of their dimension, charge, exterior characteristics and targetability to control their uptake, distribution in the body, specificity and excretion [16]. It is feasible to administer nanocarriers using varied routes like parenteral [17], nasal [18], topical [19, 20] or oral routes [21, 22]. All these advantages culminated in increasing the demand for the nanocarrier’s formulations to target multiple diseases with a broad range of properties. Dispersion of the drugs into the nanocarrier to form matrix type formulation or encaging the drug within the nanocarrier layers to give rise to reservoir type formulation are the two approaches used for carrying the payload. Ligands can be attached or adorned onto the nanocarriers’ surface for improving drugs uptake and targetability [23–25]. Nanocarriers permit accumulation of cytotoxic anticancer drugs in much higher concentration in cancer cells in contrast to normal cells. When compared to free medications, pharmaceuticals carried by nanocarriers have lower toxicity for normal tissue. Nanocarriers also offer assistance to hinder sedate degradation, reduce sedate renal clearance and amplify pharmaceutical half-life of payload within the circulatory system [6, 8].

## 8 LIPOSOME

Johnson JY invented artificial liposomes in the 1940s and obtained a patent for their application in the pharmaceutical sector. Two decades later, in the 60’s, Alec Douglas Bangham popularised liposomes by revealing the method for obtaining them and also elaborated how to use them as a biological membrane model [26]. Liposomes are spherical, closed substances made up of one (or more) phospholipid bilayer shells encaging hydrophilic core. Phospholipid, an amphiphilic molecule, is the major constituent of liposome, having a water-soluble hydrophilic head portion and a lipid-soluble, hydrophobic tail [27]. Amphiphilic nature of phospholipids provides liposomes with exceptional properties of self-sealing in aqueous solution, making it a reasonable transporter of payloads, thus finding usefulness in nourishment, makeup, horticulture and pharmaceutics. Liposomes are made up of single or many concentric or non-concentric layers having a width of around 4 nm, with an ability to swell in degree from 20 nm to several micrometers. The organization, structure and physicochemical characteristics of liposomes not only depend upon the constituent lipid’s type, size, morphology, concentration and charge but also on the solution properties *viz.* ionic strength, pH and temperature [28].

Liposomes demonstrate the advantage of being able to join and discharge two components with varying solubilities at the same time. The combination of two anticancer agents, one which is a lipid-soluble molecule (tocopherol) and another water-soluble molecule (glutathione), inside the lipid vesicle is one such layout [29]. The significant concerns related to liposomes include their low stability, inadequate drug encapsulation capacity, toxicity from residual organic solvents, and the high costs of large-scale production [30]. Fig. (**4**) represents liposomal preparation based on various loading techniques.

## 9 VESICLES FABRICATION TECHNIQUES

A few of the foremost widely used routine liposome generation strategies are being discussed below in detail [31]. The approaches used for making liposomes have one common feature. Each approach involves combining lipids with an aqueous phase in some or other way.

- **Bangham Method:** The method discovered by Bangham is even known as film hydration technique and is oldest, simplest however still habitually utilized techniques for liposome generation. The process involves dissolving lipid components in an organic solvent and subsequently removing the solvent under vacuum at temperature around 45 to 60°C. The flow nitrogen or argon stream is preferred when the required quantity of organic solvent is less under a fuming hood for the complete removal of the solvent, whereas, rotary evaporator is required while handling a large quantity of the organic solvent. Once the thin lipid film in the form of stacked bilayers is formed, then the hydration of dry lipid film is carried out using water or buffer solution; either at 60 to 7`°C or above the phase transition temperature of the lipid material. The lipid swells and gets fully hydrated during this hydration phase and thus results in the development of multilamellar vesicle dispersion which is highly mixed in dimensions and lamellarity. Preparation is required to be left overnight at 4°C to get final liposomal suspension. In this approach, the lipophilic drugs can be directly added into phospholipid at the initial stage of film formation whereas hydrophilic drugs can be incorporated into liposome during the hydration stage. Poor entrapment efficiency, problem of residual solvent and limited level generation are notable limitations of the Bangham method [26, 27].
- **Detergent Depletion Method:** This is considered a gentle approach to obtain a broad range of vesicles and liposomes with excellent homogeneity as depicted in Table **2**. The strategy used here is the creation of detergent-lipid micelles, which subsequently become rich in lipid on removal of the detergent and thus leading to the formation of unilamellar vesicles. Surfactants with high critical micellar concentration (CMC) values, like Sodium cholate, Alkyl glycoside and Triton X-100, are commonly employed detergents in this approach. Moreover, the detergent removal technique allows the formation of vesicles without ruining its pertinent natural activity; hence, it is considered a preferred method for the reconstitution of weakly soluble membrane protein [32]. Furthermore, the surfactant dialysis is found to be a substitute technique for removal of detergent. This process leads to the formation of uniform size populations of liposomes with an excellent reproducibility. However, with this process, complete removal of surfactant may not be observed thus compromising the purity of the nano formulation. Proficient surfactant removal may be possible using physical adsorption approaches [32]. While the low entrapment efficiency of lipophilic compounds and low liposomal concentration are the downsides of this method. Moreover, this method is considered to be time-consuming and the detergent removal from the preparation may also remove the tiny hydrophilic particles [33].
- **Solvent Injection Method:** This method involve injection of organic solvent-based lipid solution into an aqueous medium. Ethanol and ether are the commonly used organic solvents for this purpose. The nature of the lipid and its concentration, the ratio of the drug with lipid against the organic phase and composition of water phase are the critical factors that influence the molecule measure of liposomes created by this preparation. Batzri and Korn revealed the ethanol infusion strategy for the first time in 1973 [34]. The ethanol injection approach is quick, straightforward and repeatable and thus found to be really useful for the maker of a ready-to-use liposomal suspension. However, some lipids are less soluble in ethanol and thus it results in the formation of heterogeneous liposomes in case legitimate blending isn't obtained. The ether infusion strategy produces a concentrated liposomal product with amazing entanglement productivity. A report on inkjet method for liposome synthesis was reported by Hauschild *et al.* in the year 2005, exhibiting superior particle size control and high scaling potential as an alternative to the ethanol injection method [35].
- **Reverse Phase Evaporation Method:** This strategy of liposome preparation was given by Szoka and Papahadjopoulos in 1978 [36]. The lipids were first dissolved in organic solvent, followed by mixing of a small quantity of the aqueous phase to it and then sonicating the arrangements to form modified micelles. A rotary evaporator helps in removing organic solvent to get a thick gel, which subsequently becomes an aqueous suspension. Unfortunately, the encapsulating material is directly exposed to a harmful solvent is a major drawback of this approach, thus making it unacceptable for encaging delicate compounds like peptides.
- **Microfluidic Channel Method:** A microfluidic approach for the preparation of liposomes was reported by Jahn *et al*. in 2007 [37]. Method involves breaking a lipid stream in an alcohol (either ethanol or isopropanol) and then sandwiching it between two liquid streams in a 5–500 µm cross-sectional microfluidic channel, where blending happens at the fluid interfacing, coming about within the arrangement of liposomes. This method uses comparatively safer solvent like ethanol to give the monodisperse small liposome with uniform dimensions and distributions provided accurate control is being observed for incorporation and the fluid run rates. Processes like extrusion and/or sonication as postproduction steps are not required in this method for getting the final product. Although this method offers high adaptability, the major constraint of this method includes the use of harmful solvents and mechanical stirring that may lead to metal contamination, and the complexity involved in bulk production [38]. A lot of new microfluidic approaches have been tried in the last decades for the development of liposomes for biological and medical applications.
- **Heating Method:** Mozafari used this technique for making liposomes which involves hydrating the phospholipid components in an aqueous glycerin (3 %v/v) phase and heating the system depending on the presence or absence of cholesterol. Glycerol, being water miscible and physiologically palatable substance, is utilized since it may increase lipid vesicles robustness by controlling their aggregation and settling. Liposomes made by this strategy did not show any degradation of the lipid components, Moreover, when a temperature as high as 120 °C is used, it avoids the need for the cleansing step. The Mozafari approach, an upgraded form of the warming strategy, has as of late been utilized for the epitome and focused on the organization of food-grade antimicrobials The Mozafari approach empowers large-scale carrier framework generation in a one step and that too without the prerequisite for initial hydration of the constituent fabric or the utilization of perilous solvents or cleansers [39].
- **Emulsion Method:** This technique involves the formation of water in oil (W/O) emulsion by mixing organic phase containing phospholipids with an aqueous solution. This emulsion subsequently leads to the formation of double emulsion (w/o/w) when it is added to another water solution. Finally, the liposomal aqueous suspension is produced on the removal of organic solvent by evaporation. The emulsion method is a batch process, and hence limited volume of the liposomes can only be produced. Though liposomes of micrometric and multi-vesicular types are obtained using this technique, we need to get rid of large amounts of organic solvent from the formulation, and this restricts the use of this method [31].
- **Dense Gas Technique:** A few gases have mass transport properties that of gases and solubilizing properties like liquids and traditional solvents when handled in the region above their critical points. These gases are termed as dense gases, and their unique solubilizing property is utilized in this approach to avert the use of hazardous organic solvents. Carbon dioxide, being non-inflammable, non-toxic, non-corrosive, and cheap, is the most preferred dense gas. Moreover, it can be effortlessly removed from the formulation just by basically reestablishing barometrical pressure [40]. Thus, dense gas technique proves to be an environment friendly new formulation technique of liposome preparation.
- **Supercritical Fluid, Anti-solvent and Reverse Phase Evaporation Method:** Was successfully used to produce Single Unilamellar Vesicles (SUVs) of 20 and 50 nm size by Frederiksen *et al*. in 1994 [41]. The process involves the use of supercritical carbon dioxide as solvent, and ethanol (5–7%v/v) used as a co-solvent for solubilizing phospholipid along with cholesterol. The lipid component, along with cholesterol, is then placed in a sealed unit and carbon dioxide/ethanol is permitted to flow repeatedly through this unit. This repeated passage of solvent and co-solvent ensures complete solubilization of lipid and cholesterol; both have diverse solubilities, ultimately producing the liposomes with narrow size distribution. This lipid and cholesterol rich solution is then rapidly expanded into the drug containing aqueous phase so that entrapment of hydrophilic compound in the liposomes takes place. The frothing taking place due to rapid depressurization of the pressurized solution into an aqueous phase can be abolished by concurrent expansion and contraction of the pressurized solution using a duct prior to its dilution happening in a small pressure reprocess system [42].

In the supercritical anti-solvent (SAS) approach, lipid solution prepared using organic solvent is kept in contact with supercritical fluid, mostly CO_2_. Dissolution of the Supercritical CO_2_ in the organic phase of the solution leads to the withdrawal of hazardous organic solvent favoring the coagulation of lipid component. The precipitation of lipid components during hydration with a suitable buffer solution results in the development of liposomes. Thus, supercritical CO_2_ acts as an antisolvent for lipid and helps in the formation of liposomes. [43].

Otake *et al.* developed the supercritical reverse phase evaporation (SCRPE) method for liposome generation, and the principle involved in this technique is comparable to the decompression process used by Castor and Chu. However, both these methods differ from each other in the manner depressurization is carried out. Depressurization takes place by sprinkling the solution through a needle in case of decompression method, while in the SCRPE method, it is being carried out by releasing dense gas from the assembly cell containing the materials. SCRPE method involves mixing of the lipid, organic co-solvent and compressed gas under stirring in a variable volume cell above the lipids phase change temperature. An aqueous solution is slowly added to the mixture, and finally liposomes are produced by reducing the pressure in the cell by releasing the compressed gas [44]. Compared to Frederiksen’s method, Otake’s process of obtaining liposomes is physically simpler; it requires comparatively less carbon dioxide, and the liposomes produced are comparatively larger. However, the liposome formation mechanism is a bit intricate than the method of Frederiksen.

- **High-pressure Homogenization Method:** Method involves injecting preformed multilamellar liposomal dispersion through an orifice under a constant high pressure so that it flows with a rigid wall of the homogenizer leading to the downsizing of the liposomes [45]. Cavitation, shear stress and turbulence causes the formation of the liposomes’ structure. This method produces heterogenous liposomes as the properties and dimensions of the formed liposomes depend upon variables like pressure, temperature, and the number of cycles of homogenization for which the lipidic material is processed. Other factors that affect the outcome of this method include the initial characteristics and features of the preformed sample used (along with constitution of lipids and bulk medium) and ionic potency. Although liposomes formed using this method may still exhibit broad and variable size distribution, the major drawbacks could be the high pressure used during operation and possible contamination due to metal and engine oil [46].
- **Dual Asymmetric Centrifugation:** This method utilise centrifugation wherein a vial is rotated with a set distance and speed around the most revolution pivot. The essential qualification between dual asymmetric centrifugation (DAC) and routine centrifugation is that amid ordinary centrifugation, the vial is turned about its center (vertical hub) [47]. The vitality exchanged to the fabric within the shape of centrifuge and cavitations comes about in nanoliposomes with an estimated conveyance of 60 nm. DAC is reported to exhibit very high entrapment efficiency and is particularly found to be beneficial for manufacturing batches of less than a gram [47].
- **Freeze-drying (Lyophilization) Method:** This method is used in liposomal preparation to enhance the shelf life stability as well as to avoid the escape of the hydrophilic actives from the carriers. Generally, hydrophilic drugs when encaged in lipid-based formulations are prone to escape either during processing or storage. Even the actives may degrade due to oxidation or other chemical reactions and thus making it unsafe for consumption. The presence of moisture in the liposomal formulation could be the prime limiting factors in its commercial development and this issue can be tackled by the elimination of water (ice) from the system and that too at low pressures by freeze drying or lyophilisation [48].

The product needs to be frozen in the first step and then exposed to that scale of vacuum (<4.5 mm Hg) that is much lesser than the point where all three phases exist together (triple point) of water. Finally, one has to provide heating so that ice sublimes marking the completion of the primary drying step. Then, for desorption of unfrozen water from the product, a secondary drying under vacuum is carried out and finally dried product is taken out of the lyophilizer. Liposome products obtained from lyophilizer are extremely hygroscopic and hence are required to be sealed in airtight containers. This drying technique is best suited for thermolabile preparations that may be spoiled due to the drying by application of heat. Thus lyophilization helps in preserving various types of thermolabile biological materials (proteins, pharmaceuticals, tissues, and plasma components).

- **Membrane Contactor Method:** This technique is also known as modified ethanol injection method and was used for large scale production of the liposomes by Jaafar-Maalej *et al*. [49]. Process involves use of two pressurized containers, first for lipids dissolved in organic solvent phase and the other one for water phase. The contents of both the vessels are separated by a porous glass film with appropriate pore size to allow free movement of the organic phase. The hollow fibres of polypropylene are preferred as an alternative membrane owing to their larger cross sectional area and consistent flows. Lipid component dissolved in alcoholic phase is pushed through and extruded into water phase. Interaction amongst an organic vehicle and the water leads to the development of liposomes as lipid molecules self-assemble [50]. The new process has few notable advantages as the design of membrane contractor is simple, the liposome size can be easily controlled by tuning the process parameters and the large scale manufacturing is feasible.

## 10 NIOSOMES

Cosmetic was the first field to use Niosomes or vesicles made from non-ionic surface-active agents. These are developed by self-assembling of amphiphiles like alkyl esters/ethers and cholesterol in aqueous solutions. Niosomes are either multilamellar or unilamellar vesicles composed of sealed bilayers having water loving cavities inside and lipid loving shells outside. The evolution of nanotechnologies has witnessed the highest number of investigations focused on exploring the potential of niosomes as excellent nanocarriers. Due to their properties, niosomes have an edge over liposomes and polymersomes owing to their capacity to manipulate drugs in order to enhance their solidness and viability [51, 52]. Niosomes, vesicles composed of non-ionic surfactants, have undergone considerable developments in their manufacturing methods to enhance efficiency, scalability, and applicability. Various production techniques of niosomes are shown in Fig. (**5**). Overview of formulation methods, components, and vesicle structures in noisome preparation in Table **3**.

- **Transmembrane pH Gradient Method:** An equimolar concentration of surfactant and cholesterol is added into chloroform to get a homogeneous solution. The vehicle is then allowed to evaporate under negative pressure so as to get a thin lipid film on the inside wall of a flask or a revolving evaporator. An acidic solution (mostly citric acid) is utilized to hydrate the lipid film. The product so obtained (multilamellar vesicles) is then thawed a few times and to it aqueous solution of active was mixed under vortex mixing. The sample's pH is then increased to 7.2 with the help of phosphate buffer [53]. This technique can be successfully used to produce niosomes with 87.5 % entrapment efficiency reports Bhaskaran and Lakshmi [54].
- **Lipid Layer Hydration/Hand Shaking Method:** *A* thin lipid film is obtained in the similar fashion as described in the previous method, and the hydration of it is then carried out at the temperature slightly above the phase change temperature of the chosen surfactant using slight shaking for a particular period. The weight of each batch, position of vessel for evaporation and revolving rate of the evaporator and the rate of hydration are the crucial process variables that needs to be validated for achieving uniform product [55, 56]. Bhaskaran and Lakshmi stated excellent drug entrapment (about 78%) with this method, whereas Sathali and Rajalakshmi reported around 85 % entrapment efficiency [54, 57].
- **Reverse Phase Evaporation:** This method involves mixing ether and chloroform in an equal ratio (1:1) with the surfactant and cholesterol. Then aqueous solution of an active is mixed into the surfactant solution, and the consequential two layer system is subsequently treated for some time with ultrasound. A transparent gel so obtained is further treated with ultrasound for some more time while adding a small amount of phosphate buffer solution. The organic solvent then disappears under low pressure by evaporation and the resulting suspension is subsequently diluted with buffer solution and warmed at around 45°C in a water bath for not more than 10 minutes to produce niosomes. This noisome preparation method is reported to be excellent to encage large water soluble macromolecules exhibiting excellent entrapment efficiency compared to other methods [58-60].
- **Ether Injection:** Deamer and Bangham established this method in 1976 [61]. Method involves slowly injecting diethyl ether solution containing lipids and drug into warm aqueous phase, which is maintained at a temperature above the boiling point of the diethyl ether. This leads to the formation of large unilamellar vesicles, which are subsequently subjected for decreasing the size [62]. Entrapment efficiency of around 67.7% was achieved in the preparation of salbutamol niosomes by ether injection method [54].
- **Bubbling Method:** Is a revolutionary single step process for getting niosomes avoiding the use of harmful solvents. Three necked flask is used as bubbling unit wherein the reflux condenser for cooled water and thermometer are fixed in the first two necks, and the third neck is being used for the supply of the nitrogen. Solution of surfactants and cholesterol is prepared in a buffer at around 70 ^0^C in the bubbling unit, stirred for fifteen seconds using a high shear homogenizer, and subsequent bubbling of nitrogen gas through this solution gives rise to the formation LUVs. [63].
- **Sonication:** *B*aillie *et al*.’s approach was used to manufacture niosomes in the sonication-mediated procedure. A small vesicle can be successfully prepared using this method. Probe sonication and bath sonication are the two techniques of sonication employed for preparation in small and large volume of dispersions, respectively. Ultrasonic probe sonication Power generally varies from 20 to 200 watts, contingent upon the volume and viscosity of the dispersion [55, 64].
- **Enzymatic Method:** Enzymatic process helps in getting niosomes from a varied solution of micelles. Esterases cleave the ester links of surfactants, polyoxyethylene stearyl derivatives, leading to the formation of cholesterol and polyoxyethylene, which then combine with lipid components like dicetyl phosphate to form multilamellar niosomes [62].
- **Single Passages Method:** Micheal *et al*. patented this continuous process type technique in which the solution or suspension of a lipid is discharged through a permeable gadget and after that through a spout. Process involves the blend of homogenization and ejection at high-pressure so that uniform size niosomes can be obtained [65].
- **Micro-fluidization Method:** Is a well-known methodology for making unilamellar vesicles. In this method, surfactant and drug solution streams interface at ultrahigh speeds. The pump keeps recycling the fluid collected until vesicles of desired spherical dimensions are not obtained. Unilamellar niosomes with narrow distribution, excellent reproducibility and smaller size can be obtained using this method [66].
- **Separation of Unentrapped Drug:** Techniques like dialysis, gel filtration, and centrifugation are employed for taking out the unentrapped actives from the vesicles [55].
- **Dialysis:** Is the foremost common strategy for expelling unentrapped drugs from vesicles. In dialysis tubing, watery liposomal scattering was compared to phosphate buffer, ordinary saline, and glucose arrangement. Various specification for dialysis process using membrane is presented in Table **4**.
- **Gel Filtration:** Niosomal preparation is allowed to run through a gel filtration resin column like Sephadex-G50 and eluted into phosphate-buffered saline or plain saline to takes off the unentrapped pharmaceutical unaffected.
- **Centrifugation:** The supernatant is apportioned after centrifugation of the niosomal suspension. The sediment is resuspended to get a niosomal suspension free of unentrapped actives [67].

## 11 DENDRIMERS

Vogtle produced a novel type of polymer known as dendrimers in 1978. Term ‘Dendrimers’ originated from the "dendron" meaning tree and "meros" means parts in Greek, thus indicating the unique tree-like appearance of these compounds. After the three well-established forms of polymers *viz.*, linear or straight chain, cross-linked or ladder type, and branched or substituted linear polymers, polymers discovered by Vogtle are acknowledged as the fourth important architectural class. Throughout their 27-years of existence, they have developed significantly. Over 50, 000 patent and academic citations are already available for this significant class of polymers [68]. Structures like dendritic polymers are familiar in nature and are regularly used to improve or highlight a certain function. Dendrimers are exceedingly branched, globular, multivalent, monodisperse particles with a wide extend of applications (Fig. **6**).

A conventional dendrimer's structure is characterized by three differing highlights: a central multifunctional 'core', eras or levels of multifunctional rehashing units connected to the center, and terminal functionality or conclusion bunches. The central part consists of material (either an atom or a group of atoms), to which chains of carbon are introduced through a cycle of synthetic steps to produce a circular dendritic structure. Wide range of highly branched end-functionalized macromolecules are being synthesized by controlled manipulation of dendrimer structural characteristics are now gaining popularity due to their various potential applications [69].

Dendrimers’ synthesis resembles to molecular chemistry world by their controlled stepwise synthesis, whereas the use of monomers in the recurring structure portrays its similarity with the polymer chemistry. Although various reports explain the synthesis of dendrimers, very few of them could make it to the market. Two reasons have been identified for this failure; the first one is the multiple reaction steps involved in synthesis lead to the huge wastage of expensive starting materials, and secondly, multiple synthesis steps multiply the chances of getting structural flaws. Hence, the synthesis of dendrimers is reported to be time-consuming and expensive affair. Formation of dendrimers by various approaches is depicted in Fig. (**7**).

- **Divergent Approach:** This is the first method that was proposed for dendrimers synthesis, and it is still widely used. Stimulation or alteration of the central part or core is the primary step involved in the divergent process followed by the first monomer coupling to the core and thus leading to the first generation of the dendrimers. Stimulation of first generation (G1) ensures its reaction with other branched monomers, which leads to the formation of next generation (G2), and then similar steps are followed till we reach the desired size of the dendrimers. Creation of a new layer of branching unit leads to the formation of new generation, *i.e*., the number of branched layers attached to the core conveys the number of the generation in the dendrimers. Although this dendrimers synthesis approach usually directs the formation of highly homogenous molecules; recently various researchers have successfully applied this method for synthesis of heterogeneous dendrimers which has several types of functional group attached to the central atom [70]. In 1980, PAMAMs were the first starburst dendrimers prepared by this method. This strategy has several advantages over other strategies as it has the capacity to change the morphology of dendrimer atoms by modifying the active groups at the furthest layer as shown in Table **5**.
- **Convergent Approach:** In 1990, Hawker and Frechet produced convergent approach as an alternative method for dendrimers synthesis wherein the synthesis starts from the exterior of the surface and not from the core of the dendrimers [71]. Like divergent approach, this technique too involves the reiteration of the pairing and stimulation steps to achieve the expected dendritic structure. Initially, two groups on the surface are joined to a simple molecule to form the primary dendritic part or zero dendron generation. The activation of this fragment for reaction with other monomers is the second step that leads to the formation of dendritic wedge, also known as first generation dendron. Similar synthetic procedure is then repeated a number of times to give higher generation dendrons, and all of these are further joined to a multifunctional central atom in the final step to get the final dendrimer.

In convergent synthesis, highest purity dendrimers are synthesized as greater structural control can be achieved in this synthesis approach owing to comparatively lesser number of pairing reactions at each growth stage. Precise check over atomic weight and the creation of dendrimers with capacities in exact areas and numbers is the obvious reward of this method, whereas the difficulty of synthesizing the dendrimers in sufficient quantities is a significant constraint [72].

The most crucial challenge faced during the preparation of a higher generation dendrimers, either by any of the approaches discussed above is to avoid the formation of deficiently formed branches, and it can be successfully tackled by ensuring the addition of new generation only after the completion of the previous reaction step [68]. Various novel accelerated approaches of dendrimers synthesis have been developed in order to reduce the amount of synthesis steps, the synthesis time, the initial materials and the expenditure of the synthesis. These techniques are being discussed in the following section.

- **Hypercore or Double-stage Convergent Method:** This strategy involves pre-assembling of oligomeric species to speed up the formation of dendrimer generation. Compared to the convergent synthesis approach, this dual stage process or hyper-core decreases the steric hindrance and helps in achieving greater generations and homogeneous dendrimers [73]. This method facilitates the development of dendrimers with chemically distinguishable outer and internal branches owing to the fact that it uses two diverse types of monomers for dendrons and the hyper-core synthesis.
- **Double Exponential Growth:** Moore *et al*. introduced this breakthrough approach of dendrimers synthesis in 1995 [74]. Technique involves formation of monomers from a single starting material by divergent as well as convergent approach and coupling of these two monomers to give second generation dendron. Replicating the selective stimulation and joining steps allow the formation of a higher generation dendron. At the final stage, stimulation of the central position of the dendrons and coupling it to a multifaceted heart lead to the development of a true dendrimer. Fast blend and flexibility make this approach most versatile for dendrimers synthesis. [75].
- **Hypermonomer or Branched Monomer Approach:** Approach generates the monomers having an elevated number of functional groups as compared to conventional simple molecule and hence it is called Hyper-monomer or branched monomer approach. Dendrimers with a greater number of functional groups can be attained in minimum stages using these XY4 or XY8 hyper-monomers. The capacity to produce high-generation dendrimers in the minimum steps is the obvious advantage of this approach [76].
- **Lego Chemistry:** Different ways have been examined by researchers in order to streamline the dendrimer union strategy; Lego chemistry is one of the yields of these investigations. The amalgamation of phosphorus dendrimers has utilized Lego chemistry, and it depends on the utilization of exceedingly functionalized centers and branching monomers. The basic manufacturing strategy has experienced multiple refinements, resulting in a refined approach that could increase the terminal surface bunches almost five-fold in a single step. Apart from the capacity to extend the number of terminal surface bunches in a limited number of responses, this approach has the obvious advantage of requiring very little amount of the solvent and thus resulting in most pure final product. This method also gives rise to harmless and naturally favorable secondary products (water and nitrogen) [77].
- **Click Chemistry:** Kolb *et al* (2001) proposed this technique of preparation of dendrimers wherein smaller monomers are connected by hetero atom bonds [78]. Classic reactions applied during the synthesis of dendrimers using this approach include 1, 3-dipolar cyclo-additions, nucleophilic switch for ring opening of strained electrophilic heterocycles, and its insertion to many carbon-carbon bonds. Basic and simpler reaction settings, quickly accessible reagents and a safe vehicle are the notable advantages of this approach. PAMAM dendrons fortified with Azide functional group were produced by convergent method using click chemistry [79].

## 12 NANO-CAPSULES

Nano-capsules are the representatives of the category of nanoparticles that are formed using single or many actives that are dissolved either in aqueous or oily liquid phase and is being confined by the protective polymeric wall. This protective matrix is made up of natural or synthetic polymer and it helps in encaging the aqueous or oily material known as core inside it [80]. Nanocapsule walls are characteristically formed on the round interface of the particles or globules or even by self-assembling of the amphiphilic material. Nano-capsules, exist in submicron size (10 to 1000 nm), and upon intravenous administration, it reaches the intended target to release the encaged drug. Nano-capsules have gained interest in drug delivery owing to their key positives like sustaining the drug release, enhanced drug selectivity and effectiveness, improvement in the bioavailability of the drug and minimizing the drug toxicity [81].

Preparation of nano-capsules is being carried out by the various methods (Fig. **8**). It is the drug delivery system that can be used for numerous drugs and can be delivered by varying routes of administration with pH range varying between 3.0 to 7.5 [82].

- **Interfacial Polymerization Method:** this approach was being reported for the first time in 1989 by Fessi and Puisieux [83]. The basic concept involved in this method is to use an o/w or w/o emulsion as a template for the development of a globular membrane. Initially, oil and the actives are taken in an appropriate solvent, and this organic phase is then syringed into an aqueous phase. Polymeric film forming material, depending upon its solubility, is dissolved either in organic or aqueous phase. Addition of suitable surfactants helps in increasing the nano-capsules stability. Finally, organic solvent is removed using diffusion or evaporation process to get the aqueous suspension of formed nano-capsules [84]. This method can facilitate the production of nano-capsules with a void core, utilizing an organic vehicle in place of oleic oil, which is subsequently evaporated after the formation of the nano-capsules [85]. The characteristics of the obtained nano-capsules depend upon the concentration of the polymer, way of injecting the organic phase, proportion of organic to aqueous phase and also on the type of materials to be encapsulated [86, 87]. Fig. (**9**) shows the common nano-capsules advancement by the interfacial polymerization.
- **Nano-emulsion Template Method:** Novel high energy instruments have been introduced to get pre-form nanoemulsion, which further formulate into the nano-capsules either by using organic vehicle diffusion/evaporation or simple molecule precipitation. Organic vehicle as well as water, is mixed in the presence of a suitable surfactant using either using sonication or homogenization to get the nanoemulsion [88]. During the nanoemulsion development process, self-assembling of the surfactants at the interface leads to the reduction in the tension at the interface and thus helping to get a stable state. Depending upon the requirement of the nano-capsules to be formed, one has to either dissolve or suspend the encapsulating materials, actives, oil or any other functional substance into the internal phase or external phase [89].
- **Emulsion Diffusion/Evaporation Method:** This is one of the most commonly used techniques for getting the polymeric nano-capsules from nanoemulsion. The general procedure for developing nano-capsules using this process involves emulsification of the two immiscible phases (organic into an inorganic phase) and subsequent organic solvent elimination either by distribution into the outer phase or evaporation. Nano-capsules are produced by an amalgamation of aggregation of polymer, and interaction at the boundary of two phases during dispersion or evaporation procedures. The prerequisite for the material used to get polymeric nano-capsules involves its excellent solubility in organic solvent and at the same time its exceptional water miscibility to ensure easy elimination of organic solvent just by diffusion into water [90]. Fig. (**10**) shows up the by and large nano-capsule creation approach utilizing the emulsion-diffusion/evaporation methodology.

High encapsulation efficiencies (generally 70%), exceptional reproducibility, ease of large volume production and simple technique (homogenization not required), giving nano-capsules with narrow size distribution are the prominent advantages associated with this technique. Disadvantages reported include the difficulty in elimination of high volumes of water from the suspension and the possibility of the seepage of hydrophilic drug into the saturated-aqueous external phase during emulsification [91].

- **Emulsion-coacervation Method:** This is another technique that is based on nanoemulsion template approach for nano-capsules preparation. In emulsion coacervation process either material coacervation or substance cross-linking is used for fabricating the polymeric shell [92]. This method can be used for nano-capsules fabrication from materials having multiple ionisable groups or macromolecules holding functional groups that can be cross linked as shown in Table **6**.

In physical coacervation strategy, an electrostatic interaction between oppositely charged polymers (chitosan and pectin) is being used to prepare polyelectrolyte nano-capsules. Chitosan, being a cationic macromolecule, reacts with oppositely charged pectin into nanoemulsion and eventually gives rise to polymeric shell of nano-capsules. It has been reported that polymeric shell obtained by cross-linking using appropriate chemicals offers more stable nano-capsule system as compared to physical coacervation [93].

- **Double Emulsion Method:** This method involves emulsification of preformed nanoemulsion into a third phase (Oil or Water) to get either W/O/W or O/W/O depending on the phase order. Selection of appropriate surfactants that can provide excellent interaction between the internal and external emulsion is the critical step involved in the double emulsion method [88, 94]. In this technique, either dispersion- evaporation, precipitation or amalgamation of these techniques are used to get the polymeric shell [95, 96].

Dual emulsion (W/O/W) is widely applied for encapsulation of water-soluble drugs in the nano-capsules in order to sustain its release. Furthermore, nano-capsules formed by this technique observed to provide an excellent encapsulating platform not only for water insoluble, but also for water soluble substances concurrently [97-99].

- **Layer by Layer Method:** This technique for making multilayer nano-capsules has been recognized as a sensible technique wherein electrostatic attraction is a basic mechanism involved in nano-capsule formulation. These attractive forces lead to the deposition of polycations and polyanions on the core made up of inorganic material, followed by surrender of the template core. The trapping of the actives inside the polymeric nano-capsules, which is devoid of the core, is achieved after nano-capsule development by diffusion of water-soluble actives and electrostatic interaction with hydrophobic substance [100]. In case of Layer by Layer (LBL) method of nano-capsules preparation, varying the constitution and width of encapsulating material wall can help to regulate the targeting and releasing characteristics of nano-capsules [101]. LBL method has been recently tried in combination with nanoemulsion process utilizing o/w nanoemulsion as model core for the development of polyelectrolytes nano-capsule. Water insoluble materials may be successfully encumbered by solubilizing it in the suitable organic vehicle of nanoemulsion [102]. Cationic or anionic surface-active agents are regularly utilized to offer a charged interface for the buildup of polyelectrolyte layer in the nanoemulsion development [103, 104]. Nano-capsules prepared by LBL technique have multiple layers which could be advantageous for carrying different payload at different positions at the same time and moreover, the nano-capsules formulated using LBL method can be used to achieve varied drug release pattern in diverse pH environment just by reshuffling of the layer order [105–109].

## 13 CARBON NANOTUBES

Iijima developed carbon nanotubes (CNTs) for the first time in 1991 [5]. CNTs are carbon allotropes wherein graphene sheets are coiled-up to form a cylindrical structure called as a single or one-walled nanotube (SWNT) whereas when multiple coaxial cylinders are positioned one inside second, multiwalled carbon nanotubes (MWNTs) are formed. CNTs are long and lean graphene sheet cylinders that are obtained by the various methods as shown in (Fig. **11**).

Iijima conducted TEM microscopy analysis, in helium atmosphere, of the samples obtained from arc discharge and reported the pictures of interesting unfilled cylindrical structures with nanosized diameter. These hollow tube-like structures are called CNTs, and since then, these materials are constantly researched upon by various researchers from varied scientific fields. Research scientists from Russia around 1950 and later Oberlin *et al*. in 1976 published the first reports about these materials [110, 111]. CNTs are of great importance in every field of engineering as they possess exceptional mechanical and electrical properties owing to their varied chirality’s.

- **Arc Discharge Method:** Krätschmer *et al*. in 1990 synthesized a novel solid form of carbon, C_60_ molecule or fullerene, consisting of somewhat disordered soccer-ball shaped hexagonal packing using arc discharge method [112]. This process involves passage of an electric current (50 amps) in between graphite electrodes kept in a chamber containing an inert gas (either helium or argon) at around 50 to 700 mbar pressure. The varying potentials are then applied at both the carbon electrodes. The anode is then moved towards the cathode so that a curve appears, and then electrodes are kept at that distance (around 1 mm) till the completion of the process (around one minute). Nanotubes, along with their by-products, can be collected after releasing the pressure from and cooling of the chamber. CNTs mostly get deposited on the cathode. Addition of metals like Cobalt and Nickel or similar metals to the anode leads to formation of SWNT. However, this process can be tuned to produce primarily MWNTs using electrodes contaminated with metal catalysts like Nickel, Iron, Molybdenum, or Cobalt. Breakdown of the gaseous molecule into carbon is being catalyzed by these metals and then CNTs having these metal particles at the tip starts to grow [113, 114]. Although this is an easiest production technique to obtain best quality CNTs [115, 116], it suffers from a major constraint of moderate yield of CNTs at large-scale production level. To overcome the said drawback, Ebbensen Ajayan reported a modified standard arc discharge method for production of MWNT on huge scale [117]. The concentration of carbon vapor and its suspension in non-reactive gas, the reactor heat, the catalysts make, the incorporation of promoters and the availability of hydrogen are the notable physicochemical parameters that influence the nucleus formation and the expansion of the nanotubes, their inside and outside diameter and the type (SWNTs, MWNTs). The inclusion of pure hydrogen in this reaction diminishes the final yield [118].
- **Laser Ablation Method:** This CNT manufacturing technique was used by Smalley *et al*. in 1995 at Rice College to get the purest form of MWNTs and SWNTs [119]. Carbon object kept in a tubular furnace at very high temperature (around 1200°C) is ablated by intense laser pulses. Non-reactive gas is allowed to flow from end to end of the enclosure to carry the grown CNTs to the copper receiver. Finally, the CNTs and the derivatives like C_60_ and amorphous carbon are collected after complete cooling of the enclosure. This process can also be modified to yield either SWNTs or MWNTs by manipulating parameters like wavelength of the laser, its power and duration, heating temperature inside furnace, and composition of carbon object. Generally, a low ratio of metal to graphite in the object and a typically ≈ 1200°C furnace temperature yield superior quality SWNTs crystals while large quantity of metal gives rise to MWNTs. Crystallinity of the CNT walls is observed to be compromised if the furnace is operated at lower temperatures

Guo *et al*. came up with a pulsed laser-ablation process for the fabrication of SWNTs, and then Smalley *et al*. in 1996 successfully utilized Guo’s process of “mass production” of SWNTs [120, 121]. Other manipulation like the use of dual ray laser, is reported by Kröckel *et al*. [122] and Rao *et al*. [123]. Compared to the arc process, nanotubes obtained by laser ablation technique have around 90% purity, and their arrangement is also better graphitized. The disadvantage reported for this process is the little carbon deposit and the technique supports the growth of SWNTs. MWNTs can also be generated provided we employ special conditions for carrying out the reaction [120, 121].

- **Chemical Vapor Deposition:** CVD was proposed as a strategy for mass fabrication of carbon nanotubes in 1993 by Yacaman *et al*. [124]. It was quite evident from the outcome of arc discharge method that it was only capable of producing nanotubes in large quantities with low purity, and hence considerable efforts were being aimed at developing the manufacturing processes that could provide a regulated approach for synthesis of CNTs. CVD came up as the category of process that presents the best possibility of obtaining a controllable nanotube with predefined properties [125]. CVD is presently the foremost broadly utilized strategy for creating MWCNTs as shown in Table **7**.

CVD involves catalytic degradation with the help of a transition metal of hydrocarbon or carbon monoxide reservoir. This test is generally conducted at atmospheric pressure in a stream furnace. The catalyst to be used is kept in an earthenware or quartz vessel, which is then placed into a quartz pipe. The resulting blend comprising the hydrocarbon and non-reactive gas is permitted to flow over the catalyst bed at appropriate temperatures. The organization is brought back to normal temperature. The perpendicular furnace design is generally preferred to achieve continuous bulk manufacturing of CNTs [126].

The operating settings like the heat and the operating pressure, the type, quantity and amount of hydrocarbon, the characteristics, dimensions and the prior treatment of metallic catalyst, the type of support and the reaction time, all these factors affect the nature of the CNTs formed by CVD process [127]. Changing the actives on the exterior of the catalyst, the nanotubes dimensions can be regulated. The tube length obtained using CVD banks upon the synthesis time, and one can get tube length up to 60 mm using this process [126]. These days, the catalytic chemical vapour deposition process is regarded as the single technique for high scale CNT production that is financially viable. CVD method is being utilized to produce varying structural forms of carbon. This method permits expansion of CNTs in varied types such as fine particles [127, 128] and aligned forest of CNTs [129, 130].

## 14 POLYMERIC MICELLES

Bader *et al*. for the first time in 1984 proposed polymeric micelles as possible drug carriers for poorly soluble drugs [131]. Polymeric micelles [1] or association colloids is a nanocarrier portrayed by a central core and outer shell arrangement that originates from self-assembling of amphiphilic molecules in water. Self-assembling of amphiphilic polymers takes place at a particular concentration known as Critical Micellar Concentration or CMC. PM presents an internal lipophilic core wherein hydrophobic drugs can be easily solubilised and an external hydrophilic corona that grants prolonged blood circulation time to it [132, 133]. Fig. (**12**) depicts the structure of PM formed from different polymers.

Compared to other nanocarriers, preparation of PM is extremely simple, and its scale up is easy and feasible [134, 135]. Moreover, the amphiphilic materials utilized for the development of PM exhibit excellent interaction with bio-membrane and are also able to inhibit the efflux pumps [136, 137]. This unique property of PM, along with its extremely small size, endows it an excellent passive tumor targeting ability, even in the poorly permeable solid tumors, along with achieving the efficient cellular internalization of cytotoxic agent [132, 134]. PM, owing to all the aforesaid advantages, is considered a modern tool to tackle the issues related with the drug administration like solubility and permeability.

- **Direct Dissolution Method:** This is considered as simplest and easiest technique to prepare PM wherein the surfactant and the active, added in excess, are dissolved in water. The amphiphilic copolymer, owing to its inherent property self-assemble at or above CMC, encaging the active and leading to the formation of drug loaded PM (Fig. **13**). Process assumes the hydrophilic nature of the active as well as an amphiphilic copolymer and has a limitation of a little entrapment of active; however, this could be averted by heating the system [138, 139].
- **Dialysis Method:** This is also known as the indirect dissolution method, wherein water insoluble copolymer is first solubilized in an organic solvent which is water miscible. The solution is subsequently taken into a dialysis bag, which is placed into the beaker containing deionized water. The organic solvent and water can freely move across semi permeable membrane, thus producing the micelles. The dialysis is used for drugs having poor aqueous solubility and generally takes more than 36 hours for proper drug entrapment. Moreover, the drug loading and the size of micelles formed depend upon the organic phase used [140-142].
- **Freeze-drying:** The prerequisite for the successful implementation of this technique of preparation of PM is the selection of organic solvents such as tertiary butanol that can be of freeze dried. Initially, the solution of amphiphilic copolymer and the drug is prepared in a mixture of water/tertiary butanol, and this preparation is then lyophilized for removal of the solvents. Finally, the resultant powder is redispersed in a suitable solvent to get the micelle. Lyophilized polymeric micelle formulations found to exhibit enhanced long-term stability for intravenously administered preparations; although the presence of the residual solvent is the notable drawback of this technique [143-145]. Cryoprotectants are essential in the freeze-drying (lyophilization) process of pharmaceutical materials [1], especially for maintaining the structural integrity, stability, and bioactivity of delicate molecules such as proteins, peptides, and vaccines.

- Mitigating ice crystal formation
  - Cryoprotectants inhibit the proliferation of ice crystals during the freezing process.
  - This alleviates physical stress on the medicinal substance, safeguarding the structural integrity of active components.
  - Illustrations: Glycerol, ethylene glycol, propylene glycol
- Preserving the stability of biomolecules
  - Proteins and enzymes are vulnerable to denaturation and aggregation during freeze-drying.
  - Cryoprotectants stabilize these molecules by engaging in hydrogen bonding or other non-covalent interactions.
  - Examples include sugars such as sucrose, trehalose, and mannitol.
- Mitigating osmotic stress
  - Cryoprotectants reduce the freezing point of the solution, facilitating a more progressive freezing process.
  - This mitigates osmotic shock, which could otherwise compromise the molecular integrity of the plasma membrane.
  - Examples include polyethylene glycol (PEG) and dimethyl sulfoxide (DMSO).
- Maintaining amorphous condition
  - Certain cryoprotectants facilitate the formation of an amorphous, glass-like structure during the drying process.
  - This phase inhibits solute crystallization, maintaining the uniform distribution of the medication or active ingredient.
  - Illustrations: Trehalose and sucrose.
- Improving reconstitution
  - Cryoprotectants enhance the solubility and facilitate the reconstitution of the lyophilized product.
  - This is especially crucial for injectable or orally disintegrating formulations.
  - Illustrations: Mannitol with lactose.

- **Oil-in-water Emulsion:** This micelle production technique involves preparation of an emulsion is by adding a volatile organic solvent, which is water-immiscible (chloroform) to the aqueous medium containing the copolymer and the drug. The micelle formation takes place on vigorous shaking of the prepared emulsion and subsequent solvent evaporation. However, removal of the free active and the harmful solvent from PM remains the biggest hurdle for the success of this method [140].
- **Solid Dispersion:** This method of PM preparation is also known as solution casting. This method can only be used when both (copolymers and drug) are hydrophobic. A suitable volatile solvent is chosen that can dissolve the copolymer as well as the drug. The vehicle is then evaporated from the solution of the drug and copolymer by heating to get the drug loaded polymeric matrix. Hot water is subsequently added to the drug loaded polymer matrix to get the desired nanocarrier [146].
- **Solvent Evaporation:** Method involves the preparation of the solution containing the drug and the copolymer in a volatile organic vehicle or a blend of solvents. The organic vehicle is subsequently evaporated in order to get a thin layer on the sides of the container. Reconstitution of this dried layer with water either by sonication or stirring leads to the formation of PM. This method is also termed as film hydration technique, and it exhibits high entrapment efficiency of poorly water soluble drugs. Generally copolymers with low HLB values are preferred for preparing PM with this method [147].
- **Co-solvent Evaporation:** A combination of solvent evaporation and dialysis method may help to defeat the drawbacks associated with separate preparation techniques used for the preparation of PM. In order to manipulate the self-assembly conditions, achieve excellent entrapment of actives and meet the average size of the PM then one has to be vigilant about the choice of the solvents, the proportion of organic to aqueous phase and/or the phase addition sequence [148].

## 15 SOLID LIPID NANOPARTICLES

These are drug carrier systems in a colloidal size range (1-100nm) made up of a lipid that softens at high temperature (core) coated by fluid surfactant. The core of SLNs is water insoluble, into which the drug is either dispersed or dissolved and is then coated with a phospholipids single layer. SLNs were discovered in 1991 to offer compatibility in bio system, enhance shelf steadiness and to avoid the decomposition of encaged actives [149]. SLNs have an edge over various traditional colloidal carriers owing to their unique features like they causes little harm, huge surface area, long-lasting drug liberation and excellent cellular uptake as compared to. SLNs exhibit the capability to enhance the drug's solubility and bioavailability [150, 151]. Formation of SLNs by the various methods is shown in Figs. (**14**) and Table **8**.

- **High-pressure Homogenization:** Müller and Lucks used this process for the first time in 90s for SLN production [152]. In the HPH process, the lipid is warmed roughly 5-10 °C higher than its liquefying point and active is solubilized in it. The second step constitutes the dispersion of the drug lipid melt in a warm aqueous surfactant solution of similar temperature to obtain pre-emulsion. This pre-emulsion is further homogenised with a piston-gap homogeniser under extreme pressure conditions. The pressures speed up the liquid mixtures to a high speed (>1000 km/h), which creates high attrition that helps to trim down globule sizes to nano dimensions. The warm O/W nanoemulsion produced is then brought back to room temperature so that the lipid precipitates to yield SLN [153-156]. This homogenization cycle is repeated till the desired globule size and uniformity is achieved. Short processing times, effortless manufacturing, harmful solvent-less operation, and scale-up feasibility are the noticeable advantages of high-pressure homogenization [157, 158].

Hot and cold are the two homogenization approaches applied to produce SLNs and NLCs and are discussed in the following section.

- **Hot Homogenization:** Pre-emulsion produced by mixing the molten lipid containing active and hydrophilic emulsifying agent using a high shear homogenizer. The homogenizer is usually operated at 500–1500 bar pressure so as to decrease the dimensions of the emulsion spheres. Generally, five homogenization cycles are essential to achieve required dimensions of the spheres. The hot nanosized O/W emulsion formed yields SLNs upon cooling owing to the crystallization of the lipid in the form of globules [159].
- **Cold Homogenization:** This homogenization method was developed and accepted to beat some limitations linked with hot homogenization, like fast drug decomposition owing to elevated temperature and possible drug loss due to partitioning into the aqueous phase. The drug contact with heat cannot be avoided due to drug dispersion in molten wax and also the heat generated during the homogenization process. Thus, dry ice or liquid nitrogen is used to cool the drug containing melt rapidly. This quick cooling forms the homogeneous dispersions of drugs in lipid matrices, which is then crushed to form microparticles using any of the milling techniques [151, 157]. Microparticles are further taken in emulsifier containing ice-cold aqueous phase and homogenized at normal temperature to achieve the uniform distribution of actives in the lipid medium [160]. Particle sizes in the range of 50–100 nm using this technique [161].

- **Ultrasonication:** High-speed stirring or ultrasonication is another technique used to produce SLN. This method differs from above mentioned preparation method at the homogenization step. Method involves dispersion of drug containing lipid melt using a top speed shear mixer or ultrasonicator. The force of intense turbulent eddies of sonicator leads to development of a hot O/W emulsion. Finally, cooling of these dispersions yields desired Solid Lipid Nanoparticle (SLNs) and nanostructured lipid carriers (NLCs) [162, 163]. The metal contamination of the final product originating from sonicator probes is a disadvantage of ultra-sonication. Although use of bath sonication can avoid this problem, however, it needs to be clubbed with other consequential procedures [157, 164].
- **Solvent-emulsification Evaporation:** preparation of nanoparticles using this method is carried out in three basic steps. Firstly, a clear homogenous lipid solution is obtained by solubilising lipid component in a known volume of appropriate organic solvent. In the next stage, solution obtained in step I is mixed using a high-speed homogenizer with a known volume of aqueous phase to form a crude emulsion. Nanoemulsion is then obtained from the coarse emulsion in the final step by applying very high pressure using a top-pressure homogenizer. The nanoemulsion is then stored overnight under continuous stirring or placed under a fume hood to remove the residues of harmful solvent. Elimination of the volatile solvent results in the formation of a nano-dispersion due to the precipitation of lipid material in the aqueous phase. The precipitated lipid is filtered out through sintered disc filter from aqueous medium of nano-dispersion. The nanoparticles obtained using this strategy are usually nano-sized, non-aggregated (single entity) and exhibit high encapsulation ability [165]. Moreover, this method avoids exposure of the drug to high temperatures; thus, highly thermo-labile drugs can be encapsulated using this technique. The major constraints of this method are that it involves harmful organic vehicles and the dispersion produced necessitates concentrating it using more processing [166].
- **Supercritical Fluid:** The preparation of SLNs using supercritical fluid is typically a modern approach as use of any of the solvents is avoided and SLNs are obtained from gas saturated solutions. Melting of the lipid material is carried out in the gas saturated solutions, which is subsequently mixed in the super critical fluid (SCF) under pressure. The resultant product is allowed to expand by spraying through atomizer to set SCF free and produce finely desiccated lipid material. Supercritical carbon dioxide solution is commonly used for this method of SLN preparation. SCF method does not involve use of any harmful organic vehicle, and excellent lipids SCF miscibility is an added advantage of this technique [167, 168]. SLNs produced using this technique exhibits uniform particle size distribution [169].
- **Microemulsion:** Gasco used this method for preparation of SLNs before 90s [170], and afterwards it has been explored by numerous scientists [166, 171]. The basic principle involved in this method is diluting a microemulsion in chilled water results in the development of nanoemulsion and the successive agglomeration of lipid yields SLNs and NLCs. In general, active is dispersed in lipid melt at a temperature higher than the lipids softening scale, and then hydrophilic surfactant solution phase (maintained at the same temperature as that of lipid phase) is mixed under gentle stirring to form a clear and robust microemulsion [172, 173]. Product is then transferred into water, maintained at 2 to 10°C, under mild mechanical assimilation [174]. Normally, the volume of the chilled aqueous phase is much higher than that of the hot emulsion. This dilution stage leads to the instant crystallization of lipids to form SLNs or NLCs. The technique is really straightforward, reproducible and easy to scale-up.
- **Spray Drying:** It is a one-step process that gives rise to re-dispersible powder and is considered an alternative and cost-effective process to lyophilization. The process involves atomization of the drug solution or its dispersion into hot air so that rapid evaporation of solvent takes place, leading to the formation of dry solid particles. Carbohydrate or a small amount of lipid mixed with drug dispersion to protect the drug from high temperature. Mixture of ethanol water helps in reducing the melting of the lipid component [175].

There are several positives of the spray-drying technique, like it is easy to regulate and manage parameters like nature of the solvent, concentration of the solute, flow rate of solution and gas in drying chamber, drying temperature and relative humidity, atomization rate *etc*. Thus, it is possible to optimize properties of the final product in terms of dimensions, size distribution, shape, morphology and density along with the rheological properties like bulk density, flowability and dispersibility [176]. However, degradation of some macromolecular drugs due to various features like thermal impact on the globules during desiccation, high force in the atomizer and adsorption of macromolecules at the liquid/air interface of the spray solution are a few notable disadvantages of using spray-drying technique.

- **Double Emulsion Method:** Double emulsion or multiple emulsion uses three steps process for the encaging water-soluble drugs [177]. First step involves solubilising an active and a stabilizer in water and then aqueous phase is emulsified in lipids containing water-immiscible solvent [178] or in molten lipids (free from solvent) to give either water in oil emulsion or inverse emulsion [179, 180]. This primary emulsion is then mixed, under incessant stirring (either applying sonication or homogenization), with an aqueous phase of hydrophilic emulsifier or stabilizer to obtain a multiple emulsion (W/O/W), and in the final step, solvent is allowed to evaporate to form the nanoparticles. A stabilizer used in this method prevents the drug favouring to the outer aqueous phase during the solvent removal. This technique can be utilized if surface modification by insertion of hydrophilic polymers for changing nanoparticle properties is desirable; however, this method produces larger sized particles and escape of water-soluble actives to the external water phase is another prominent disadvantage of this method [181, 182].
- **Membrane Contractor Technique:** An explicit film contactor is used for SLNs development. Method involves pressing of the solid lipid, maintained at the temperature above lipids melting point, through the holes of a film to form small globules. Simultaneously, surfactant solution prepared in an aqueous phase is allowed to circulate along the opposite side of the membrane inside a contractor. This sweeps away lipid from the membrane as aqueous surfactant solution flows tangentially onto the membrane surface. The warm emulsion is then chilled down to produce SLNs [183]. Higher scale manufacturing is feasible with this method as 0.1 µm pore size membrane results in high fluxes. However, constraints of this method like complicated structure and unit is prone to blockage restricts its applications [184, 185].

## CONCLUSION

Nanocarriers are significantly advanced drug delivery systems, presenting multiple benefits over conventional methods. The ability to engineer nanocarriers with specific properties - such as size, charge, and surface characteristics - allows for enhanced targeting, improved drug stability, and reduced side effects. Liposomes, niosomes, dendrimers, nano-capsules, carbon nanotubes, polymeric micelles, and solid lipid nanoparticles each offer unique advantages for drug delivery, with various preparation methods tailored to their specific needs. Despite the progress, challenges such as stability, production scalability, and potential toxicity remains the point of concern. Techniques like the Bangham method, solvent injection, and chemical vapor deposition provide a range of options for producing nanocarriers, each with its trade-offs. Emerging technologies, such as artificial intelligence and automation have further enhanced the design and optimization of nanocarriers. Artificial intelligence driven approaches allow for the prediction of drug-nanocarrier compatibility, optimization of formulation parameters, and scaling up production processes with greater efficiency. Additionally, the integration of stimuli-responsive and multifunctional nanocarriers has opened new avenues for treating complex diseases like cancer and genetic disorders. Continued research and development are crucial to overcoming these challenges and optimizing nanocarrier systems for clinical use. The evolving landscape of nanotechnology promises to further enhance therapeutic outcomes, making nanocarriers a pivotal component in modern medicine and drug delivery.

## Figures and Tables

**Fig. (1):**
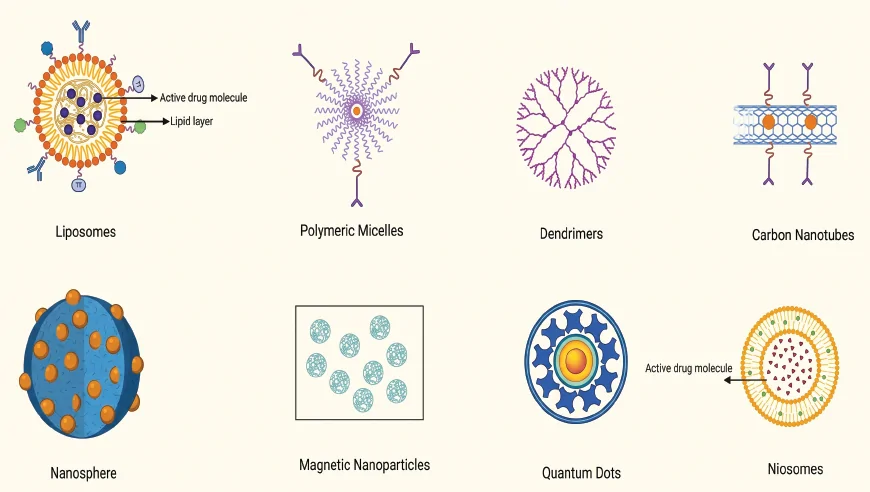
Classification of various nanocarriers-based on nanotechnology assisted techniques for fabrication. The figure prepared using Bio render AI-based software.

**Fig. (2):**
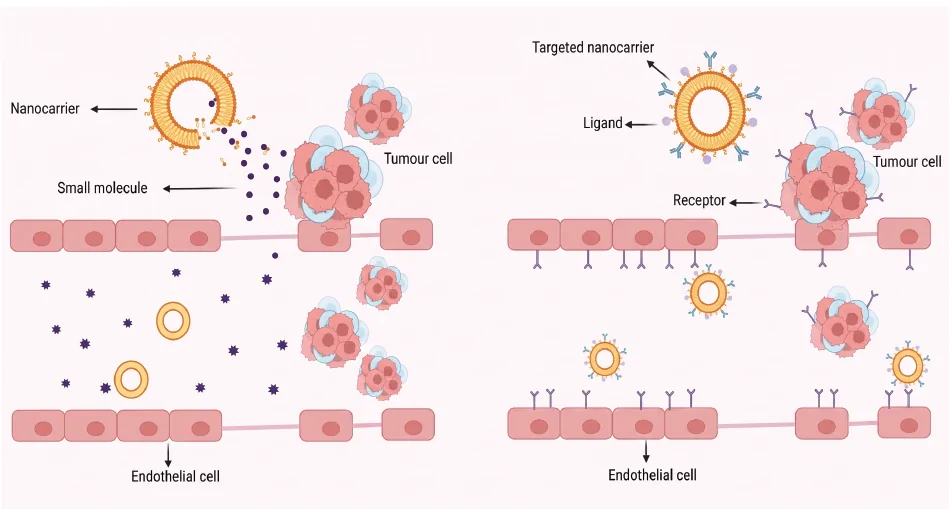
Various targeting approaches associated with nanocarriers are used for management of various complications. The figure was prepared using Bio render AI-based software.

**Fig. (3):**
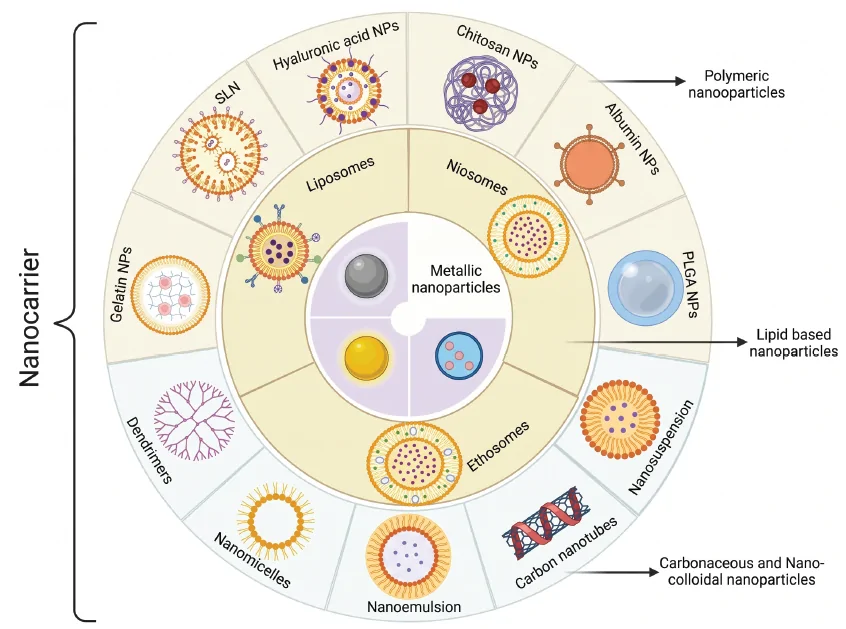
Types of different nanocarriers. The figure was prepared using Bio render AI-based software.

**Fig. (4):**
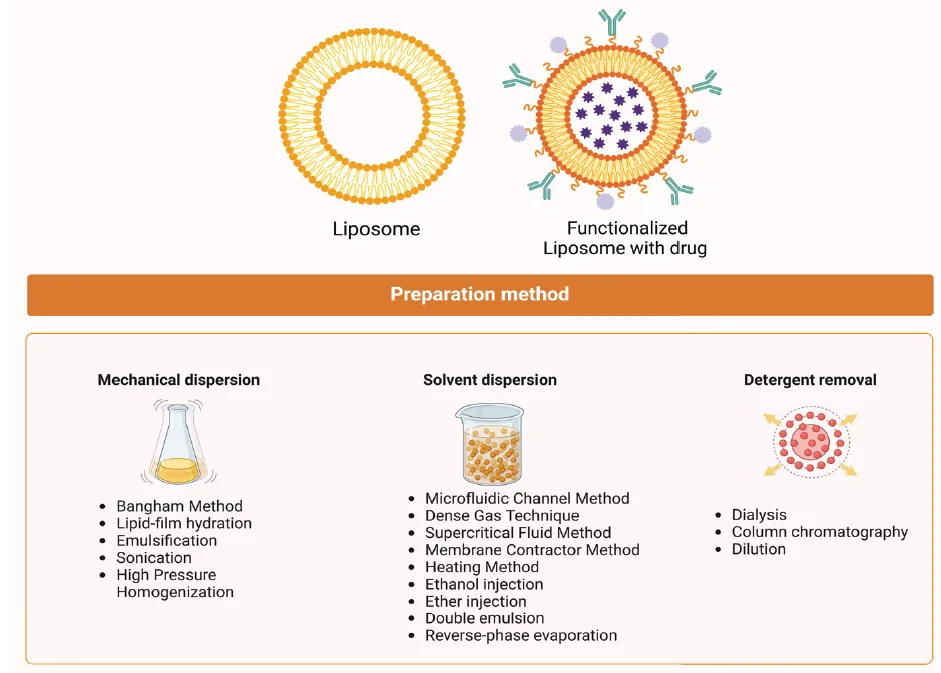
Various techniques used in fabrication of liposomes. The figure prepared using Bio render AI-based software.

**Fig. (5):**
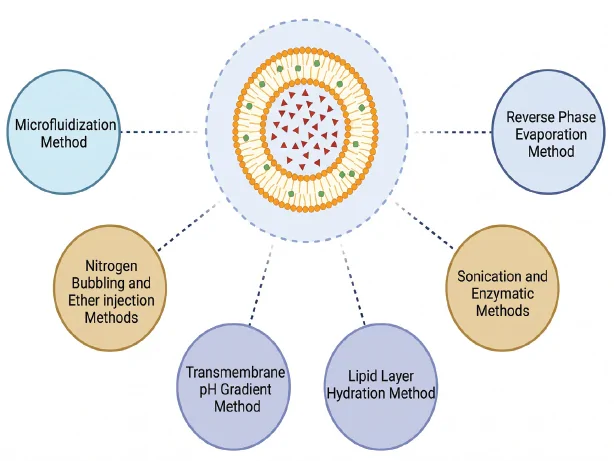
Various techniques used in the formulation of Niosomes. The figure was prepared using Bio render AI-based software.

**Fig. (6):**
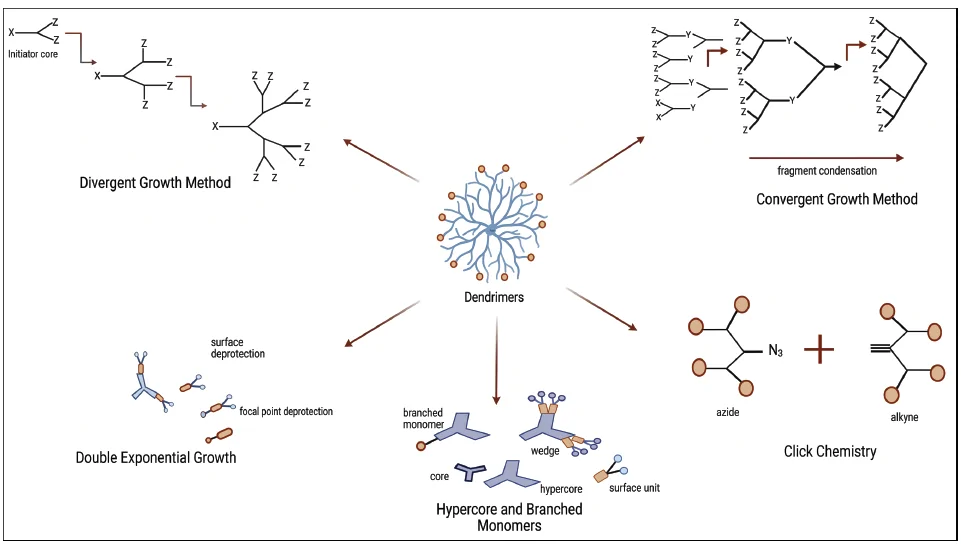
Structural identity of dendrimers. The figure prepared using Bio render AI-based software.

**Fig. (7):**
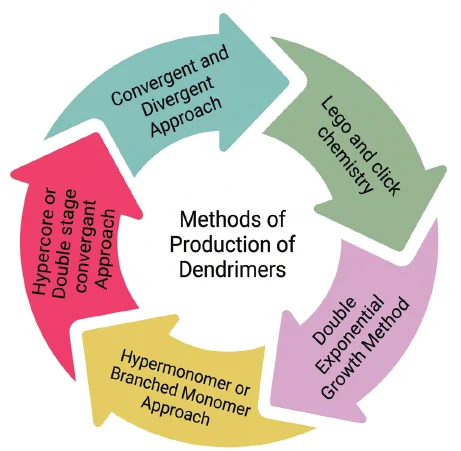
Various production techniques associated with dendrimer. The figure prepared using Bio render AI-based software.

**Fig. (8):**
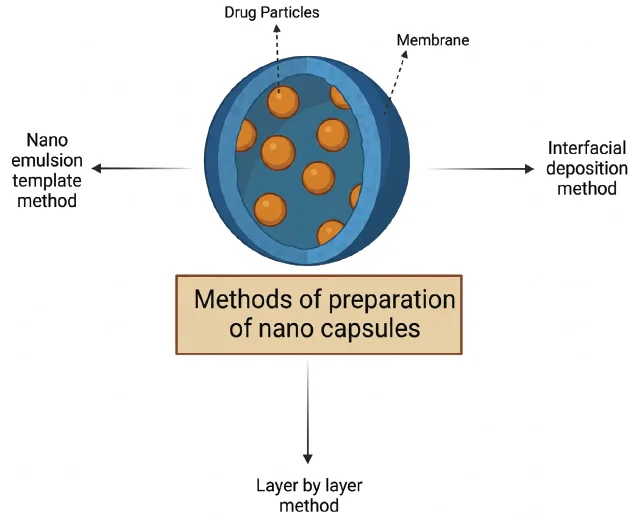
Nano capsules preparation technique. The figure was prepared using Bio render AI-based software.

**Fig. (9):**
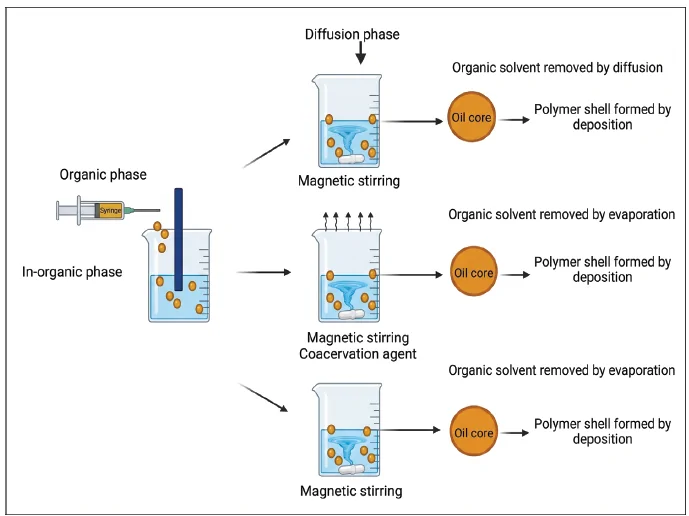
Nano capsules fabrication using interfacial polymerization techniques. The figure prepared using Bio render AI-based software.

**Fig. (10):**
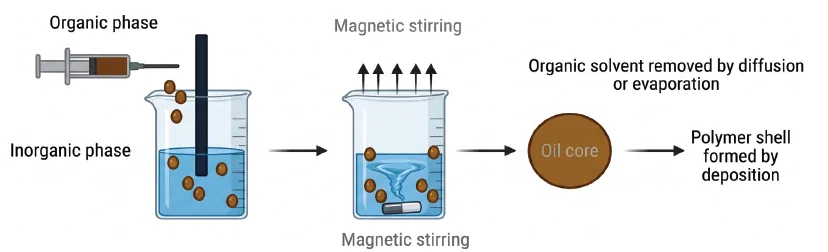
Emulsion-diffusion/evaporation methodology in fabrication of nano-capsule. The figure was prepared using Bio render AI-based software.

**Fig. (11):**
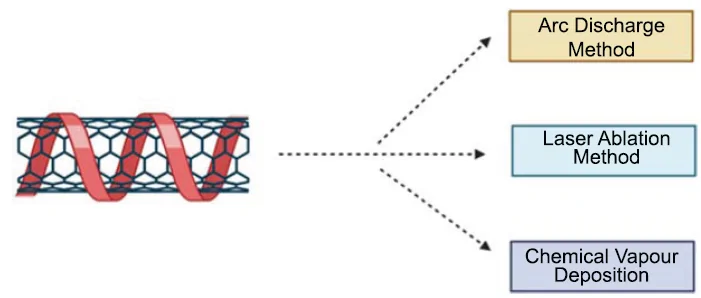
Methods of preparation of nanotubes. The figure was prepared using Bio render AI-based software.

**Fig. (12):**
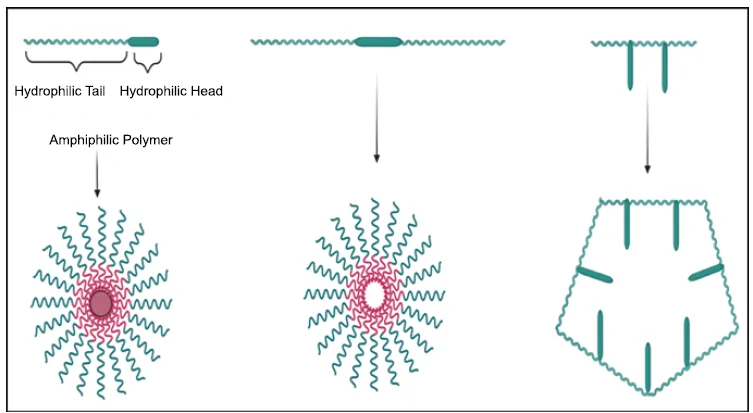
Structure of polymeric micelles. The figure prepared using Bio render AI-based software.

**Fig. (13):**
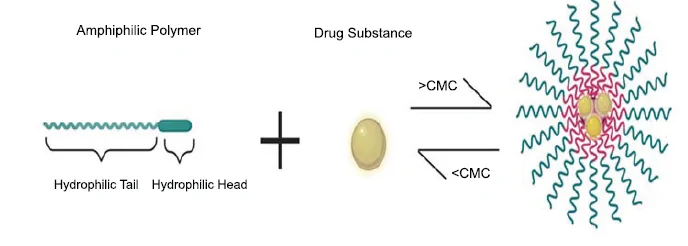
Drug loading process in polymeric micelles. The figure was prepared using Bio render AI-based software.

**Fig. (14):**
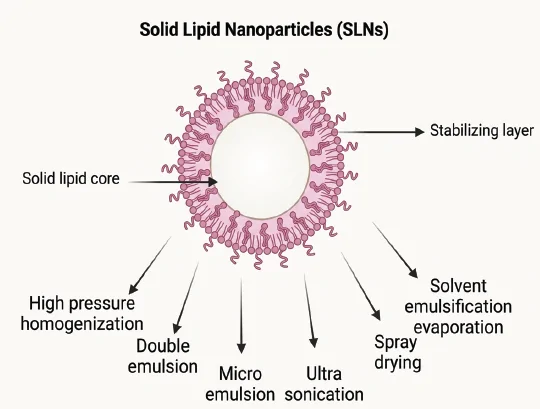
Various methods associated with preparation of solid lipid nanoparticles. The figure was prepared using Bio render AI-based software.

**Table 1:** Depicts production techniques of various nanocarriers.

**Nanocarrier**	**Synthesis Techniques**	**Properties**	**Applications**	**Challenges**
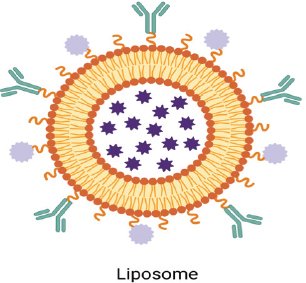	Bangham method, detergent removal method, ethanol/ether injection, reverse phase evaporation, detergent depletion method, microfluidic channel method, high-pressure homogenization, heating method, dual asymmetric centrifugation, supercritical method	Biocompatible, biodegradable, low-toxicity phospholipid bilayer vesicle. Can encapsulate both hydrophilic and hydrophobic drugs.	Effective delivery of biologically active agents, including drugs and genetic materials.	Fast clearance and off-target accumulation
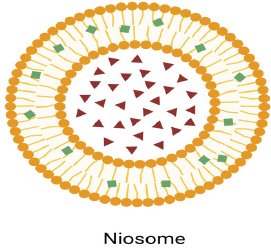	Ether injection, handshaking, reverse phase evaporation, bubbling nitrogen, transmembrane pH gradient, microfluidization, enzymatic method	Nonimmunogenic, biodegradable, and biocompatible. Controlled drug release and long-term stability with hydrophilic, amphiphilic, and lipophilic properties.	Enhancing drug stability, targeting, and maintaining active substances in circulation.	
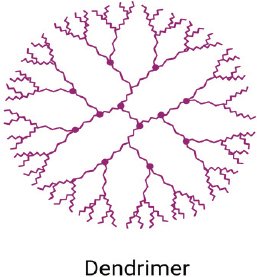	Divergent method, convergent method, double exponential growth, hypercores, and branched monomers	Hyperbranched, radially symmetric, homogeneous, highly defined, monodisperse molecules.	Photodynamic therapy, neutron capture therapy, drug delivery, liver targeting, gene delivery, and imaging applications.	Fast clearance and off-target accumulation
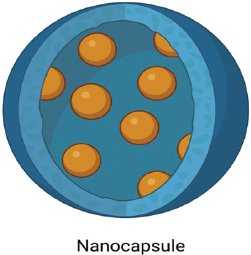	Nanoprecipitation, polymer coating, layer-by-layer method	Mini-emulsion polymerization enhances controlled release properties.	Delivery of plasmids *in vivo* for liver cells, oral, parenteral, and ocular drug delivery systems.	-
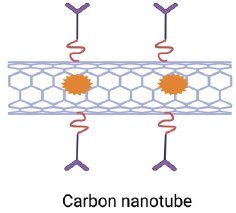	Arc discharge, chemical vapor deposition, laser ablation	Crystalline beehive pattern of allotropic carbon with unique electrical, dynamic, and elastic properties. Available in single or multi-layer forms.	Gene and drug delivery, peptide delivery, artificial implants, tissue engineering, and cancer cell identification.	Insoluble unless functionalized
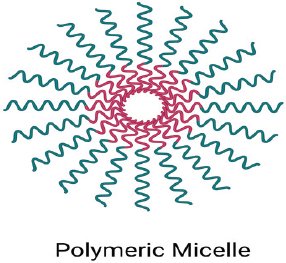	Direct dissolution, indirect dissolution	Dynamic amphiphilic molecule aggregation system with enhanced biostability.	Encapsulation of hydrophilic or hydrophobic drugs.	Relatively low drug loading capacity and may reduce the effectiveness of the conjugated drug
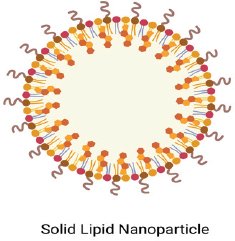	High pressure homogenization, ultra-sonification, microemulsion, solvent emulsification-evaporation, solvent injection, Solvent injection, Membrane contractor, and Phase inversion		Effective delivery of biologically active agents, including bioactive compounds and synthetic drugs	Limited drug loading capacity, potential for burst drug release, polymorphism, phase separation, difficulty in sterilization, and concerns regarding stability during storage due to possible drug expulsion from the lipid matrix as the solid lipid crystalizes.

**Note: ** The figures presented in the table were prepared using Bio render AI-based software.

**Table 2:** Various method applied in the fabrication of varied vesicular system with advantageous as carrier to deliver the drug at target site.

**Method**	**Advantage**	**Disadvantage**	**Type of Vesicles**
Bangham Method	Simple process	Difficulty in removing organic solvent, large vesicles without controlling on particle size, Time consuming & sterilization issue	MLVs
Detergent depletion Method	Particle size good, Simple process, Homogenous product	Difficulty in removing organic solvent, Detergent residue, Time consuming, Sterilization is needed	MLVs, LUVs
Ethanol/ether injection	Simple process	Organic solvent residue, readily to nozzle blockage in ether system, time consuming, sterilization issue	SMVs, SUVs
Reverse phase evaporation	Process simple, good encapsulation efficiency	The huge amount of organic solvent used, the process is time consuming, there is a sterilization issue, it is not ideal for encapsulating sensitive molecules.	MLVs, LUVs
Micro fluidic method a) Micro hydrodynamic focusing. b) Microfluidic droplets. c) Pulsed jet flow microfluidics	Good particle size control	Difficulty in removing organic solvent, not suitable for bulk production	SUVs, LUVs (for MHF), GUVs (for MD), GUVs (for PJM)
Dense gas technique	Sterilization in situ is possible, resulting in a stable and homogeneous liposome with minimal organic solvent use.	Multiple stages are required to reach the final liposome size, high pressure of up to 200-300 bar is required, and nozzles are easily blocked.	-
High pressure homogenization method	Liposomes with a diameter of up to 10 nm can be produced, and the design is simple enough for mass manufacturing.	High pressure, sterilization issues, liposome production that isn’t uniform, and organic solvent residue	Small liposome for IV injection
Heating method	No contamination (*e.g*. organic solvent) is required, and no sterilization is required.	High temperature used	MLVs, SUV
Dual asymmetric centrifugation	High trapping efficiency, simple approach, homogeneous liposome manufacturing with 60 nm size	High pressure, agitation, not appropriate for bulk manufacturing	Nanoliposomes
Supercritical method -Supercritical fluid injection and decompression -Supercritical fluid injection and decompression	- Particle size control, in-situ sterilization, and reduced organic solvent usage are all advantages. - No nozzles required, one-step production, minimal organic solvent use, quick process, scale-up potential, and improved stability	High cost, low yield, and utilization of high pressure up to 350 bars. High cost, and utilization of high pressure up to 200 bars.	SUV

**Table 3:** Overview of formulation methods, components, and vesicle structures in noisome preparation.

**Formulation Method**	**Components**	**Structure size (nm)**
Ether injection method	Cholesterol	Uni-lamellar vesicles
Sonication	Span 60 cholesterol	Multi lamellar vesicles
Hand shaking method	Span 60 cholesterol	Vesicles formation
Reverse phase evaporation	Span40, span 60	Vesicles formation
Bubbling of nitrogen	Cholesterol, buffer	Large uni-lamellar vesicles
Transmembrane pH gradient method	Cholesterol	Multilamellar vesicles
Micro fluidization method	Mono-palmitin glycerol cholesterol dicetyl phosphate	Uni-lamellar vesicles
Enzymatic method	Polyoxyethylene stearyl derivatives	Multilamellar vesicles

**Table 4:** Various specification for dialysis process using membrane.

**MWCO (kDa)**	**Pore Size**	**Typical Applications**
0.5–1 kDa	Smallest pores (~1 nm)	Separation of small molecules, peptides, and ions.
3–5 kDa	Small molecules (~2 nm)	Removing salts, small metabolites, and low molecular weight impurities.
10 kDa	Medium molecules (~3 nm)	Separation of small proteins, peptides, and oligonucleotides.
20–50 kDa	Large molecules (~5 nm)	Concentration or purification of proteins and larger

**Table 5:** Comparison of dendrimer synthesis methods: advantages and disadvantages.

**Method**	**Advantages**	**Disadvantages**
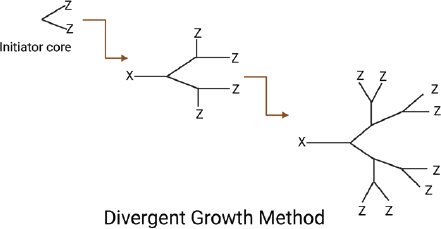	Fast synthesis; large-scale manufacturing; synthesis of highly symmetric dendrimers; surface of dendrimer can be easily adjusted with appropriate functional groups; high-generation dendrimers can be formed.	Possibility of flaws in higher generation dendrimers; difficulty in separating the desired product from reactants; excess of reagents; multiple steps are required to build a big structure; a significant quantity of starting material is required; probable incomplete reaction of the terminal groups.
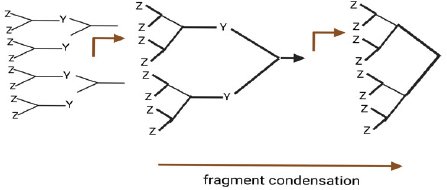	Purification of the desired product is simple; defect occurrence is low; synthesis of asymmetric dendrimers is possible; there are few reactions per molecule; structural control is better than with the divergent approach.	Low yield; difficult to change the terminal groups; does not allow for the creation of several dendrimers.
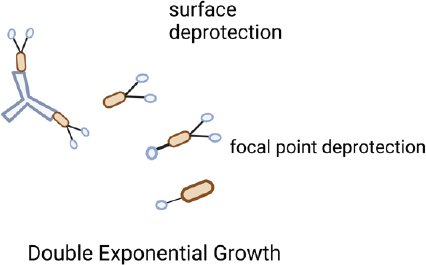	Huge multifunctional dendrons or dendrimers; symmetric, supramolecular, or asymmetrical dendrimers; high synthetic yields; several dendrimers made from the same monomers twice or three times.	Approach employs both convergent and divergent processes, the procedure is time-consuming.
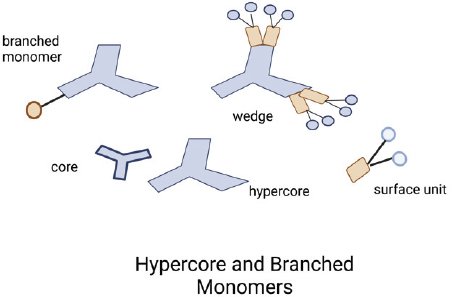	Allows for the development of high-generation dendrimers; employs a hypercore to eliminate steric effects; aids in the formation of more monodisperse dendrimers; allows for the formation of dendrimers with chemically distinct interior and exterior branches. Dendrimers with several functional groups in fewer steps; allows high-generation dendrimers to be formed in a few processes.	It takes a long time to make the hypercore, dendrons, and final dendrimers. Synthesis necessitates many growth and activation steps; the acceleration is confined to the production of dendrimers; monomer synthesis is a lengthy process.

**Note: ** The figures presented in the table were prepared using Bio render AI-based software.

**Table 6:** Overview of methods, drugs, polymers, solvents, and activities in drug formulation.

**Method**	**Drug**	**Polymer**	**Solvent**	**Activity**
Nanoprecipitation method	Indomethacin	PCLMw60kDa	Acetone	Anti-inflammatory
Emulsion diffusion method	Indomethacin	-PCLMw10 -PVA poloxamer	Ethyl acetate water	Anti-inflammatory
Double emulsification method	Insulin	PLAMw 10kDa, DCM, Sorbitan monoleate	Water	Anti-diabetic
Emulsion coacervation method	Turmeric oil	Sodium alginate Mw 80-120kDa	Ethanol, Methanol, water	Antifungal Antibacterial Antioxidant
Polymer coating method	Salmon calcitonin	Poloxamer 188	Water	Calcium regulator
Layer by layer method	Artemisin	Polycations and polyanions	Miscible solvent	

**Table 7:** Comparative overview of three key methods for synthesizing single-wall carbon nanotubes.

**Parameter**	**Arc Discharge Method**	**Chemical Vapor Deposition**	**Laser Ablation**
Discovered by	Introduced by Ajayan and Ebbesen in 1992	Developed by Endo *et al*. in 1993	Developed by Smalley in 1995
Yield (%)	30-90	20-100	~70
Single wall nanotubes characteristics	Short nanotubes Diameter: 0.5-1.5nm	Elongated nanotubes Diameter: 0.5-4.0nm	Long nanotubes Diameter: 1.0 -2.0nm
Raw material availability	Difficult	Difficult	Easy
Process control	Difficult	Difficult	Easy, can be automated
Reactor design	Difficult	Difficult	Easy
Production rate	Low	Low	High
Product purity	High	High	High
Product yield	Moderate	High	High
Process nature	Batch type	Batch type	Continuous
Cost	High	High	Low
Advantage	Inexpensive, SWCNTs have a small number of structural flaws.	Long nanotubes are produced with exceptional purity and adjustable diameter.	SWCNTs are produced in a consistent manner. There are very few structural flaws.
Disadvantage	Nanotubes of a short length. SWCNTs are of varying sizes. Purification is required for heterogeneous reaction.	Most suitable for the production of MWCNTs	Technique that is both expensive and time consuming.

**Table 8:** Overview of methods for nanoparticle formulation: drugs, advantages, and disadvantages.

**Method**	**Drug**	**Advantage**	**Disadvantage**
High pressure homogenization	Cyclosporin A, Itraconazole	Good stability Low cost, Large Scale production	High polydispersity High temperature Energy-intensive procedure
Ultra-sonification	Apomorphine, Rivastigmine	Small size of the particles High shear mixing	Metal toxicity Time-consuming process
Microemulsion	Cravedilol, Cyclosporin	Low energy consumption Avoiding the use of heat	Low return on investment Excessive surfactant use
Solvent emulsification evaporation	Piroxicam loaded SLNs, Buspirone	Low energy consumption No heating is used Particle size is small	Emulsion instability Organic solvent residue
Solvent injection	Methotrexate	Low energy input Quick production Avoiding the use of heat	Organic solvent residue
Membrane contractor	-	Low energy input Large-scale manufacturing Particle size control	Membranes are clogged
Phase inversion	-	Low energy consumption Free of organic solvents	Emulsion not stable

## LIST OF ABBREVIATIONS

CMC: Critical micellar concentration
CNT: Carbon Nanotube
CVD: Chemical vapor deposition
DAC: Dual Asymmetric Centrifugation
EPR: Enhanced permeability and retention
GUV: Giant Unilamellar Vesicles
HPH: High-pressure homogenization
LUV: Large unilamellar vesicles
MLV: Multi lamellar vesicles
MWNTs: Multiwall carbon nanotubes
NLC: Nanostructured lipid carriers
PM: Polymeric micelles
SAS: Supercritical anti-solvent
SCRPE: Supercritical reverse phase evaporation
SUV: Single unilamellar vesicles
SWNT: Single walled carbon nanotubes
